# Calcium—Nutrient and Messenger

**DOI:** 10.3389/fpls.2019.00440

**Published:** 2019-04-25

**Authors:** Kathrin Thor

**Affiliations:** The Sainsbury Laboratory, University of East Anglia, Norwich Research Park, Norwich, United Kingdom

**Keywords:** calcium, cell wall, immunity, nutrient signaling, roots, uptake, transporters, kinases

## Abstract

Calcium is an essential element needed for growth and development of plants under both non-stressed and stress conditions. It thereby fulfills a dual function, being not only an important factor for cell wall and membrane stability, but also serving as a second messenger in many developmental and physiological processes, including the response of plants to biotic stress. The perception of non-self hereby induces an influx of calcium ions (Ca^2+^) into the cytosol, which is decoded into downstream responses ultimately leading to defense. Maintaining intracellular Ca^2+^ homeostasis is crucial for the ability to generate this signal. This review will describe the current knowledge of the mechanisms involved in uptake and transport of calcium as well as cellular homeostasis and signal generation, describing known genes involved and discussing possible implications the plant’s nutritional status with regard to calcium might have on immunity.

## Introduction

Calcium is an essential macronutrient in plants, with concentrations in the shoot ranging from 0.1 to over 5% of dry wt (Marschner, 1995; White and Broadley, 2003). It thereby exhibits a dual function, both as a structural component of cell walls and membranes and as intracellular second messenger. The uptake, distribution, and storage in the plant therefore need to be tightly regulated in order to comply with both tasks.

To fulfill the structural role, calcium has to be available for the plant in sufficient amounts. In general, calcium deficiency as a result of low soil availability is not very common (White and Broadley, 2003). Deficiency symptoms occur however more often in developing tissue such as young leaves and fruits, due to low remobilization from old to young tissue via the phloem. This leads to a strong dependency on supply via the xylem and thus on transpiration, which in young tissues is not very high. Resulting diseases are for example tipburn in lettuce or blossom end rot in tomato (White and Broadley, 2003; Hirschi, 2004).

**Figure 1:**
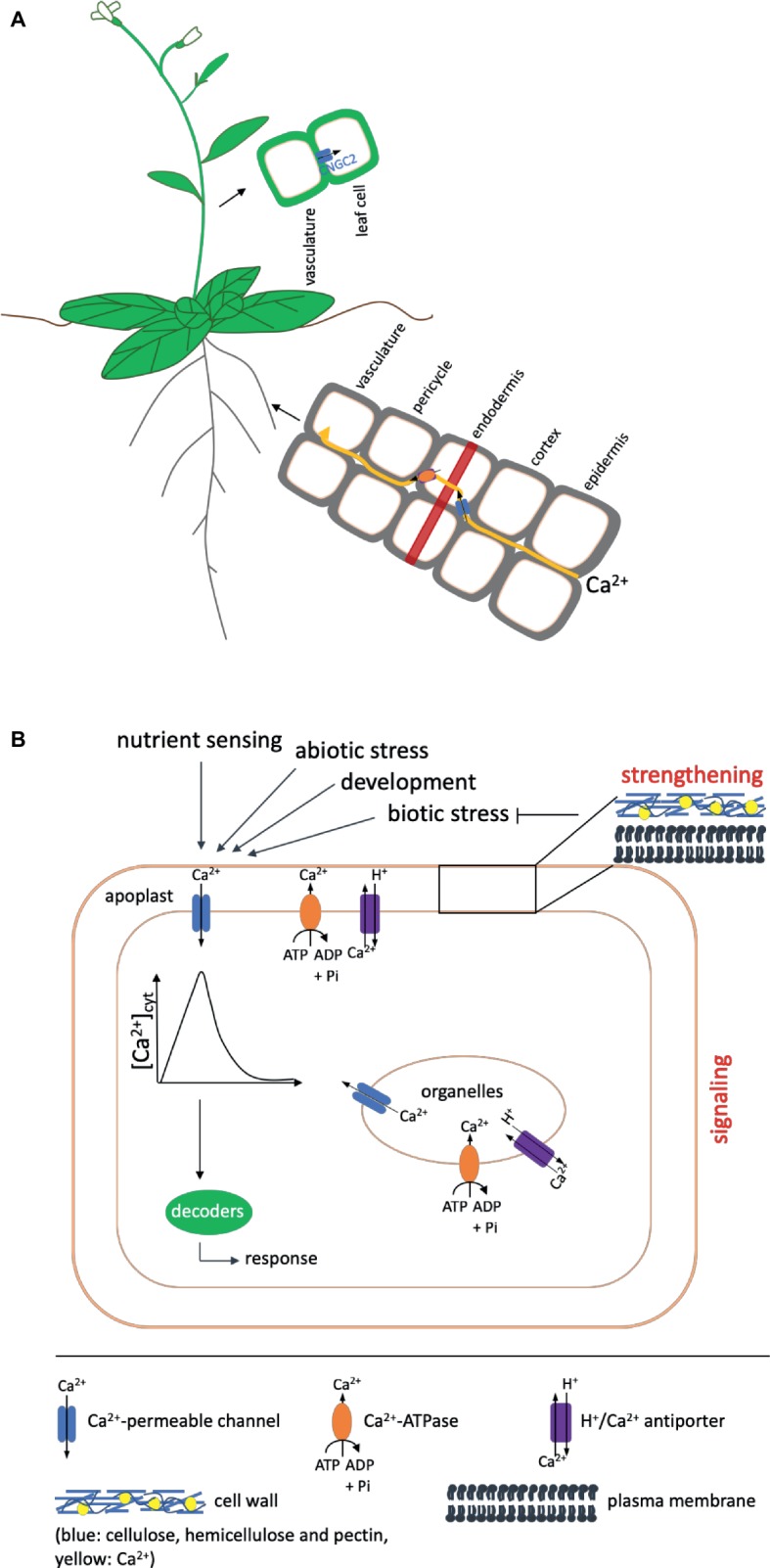
Overview of Ca^2+^ uptake and the functions it fulfills in the plant. Shown are only components mentioned in the text. **(A)** Ca^2+^ is taken up by the root and transported to the shoot in a mainly apoplastic way to avoid interference with its function as second messenger. To circumvent the casparian strip (indicated as red band), it has to enter the cytosol of the endodermal cells via channel proteins (shown in blue) and subsequently be exported into the apoplast via Ca^2+^-ATPases or Ca^2+^/H^+^ antiporters (shown in orange) (Clarkson, 1993). Influx into leaf cells after unloading from the vasculature in *Arabidopsis* has been proposed to occur via CNGC2. **(B)** Ca^2+^ fulfills two functions in the plant: It has a strengthening effect on cell walls and membranes and acts as a second messenger in signaling events during development and in response to environmental cues. A strengthened cell wall protects against pathogens (indicated by a blunt arrow). In addition, different kinds of biotic stress also induce signaling cascades, in which calcium serves as second messenger. In such signaling cascades, like in those induced by other events, Ca^2+^ enters the cytosol from compartments of higher concentration (apoplast, organelles) via channel proteins (blue) to induce an increase in the cytosolic calcium concentration [Ca^2+^]_cyt_, the Ca^2+^ signal, which is decoded by downstream components into an appropriate response. The signal is terminated by transport of Ca^2+^ out of the cytosol via Ca^2+^-ATPases (shown in orange) or H^+^/Ca^2+^ antiporters (purple) in the plasma or organeller membranes. The presence of these export proteins differs in different organelles. Details of the proteins and genes involved are given in the text. Arrows on top indicate events that induce Ca^2+^ signals. One such event for example also is the sensing of other nutrients.

Besides its structural role, the main function of calcium lies in its ability to serve as a second messenger in a variety of processes ranging from root or pollen tube growth and fertilization (Michard et al., 2011; Monshausen et al., 2011; Ortiz-Ramírez et al., 2017; Zhang et al., 2017) to responses to abiotic (Knight et al., 1991, 1997) as well as biotic stress (Blume et al., 2000; Lecourieux et al., 2002, 2005). Transient, sustained, or oscillatory rises in the cytosolic Ca^2+^ concentration thereby serve as a signal, which is decoded into downstream responses (McAinsh and Pittman, 2009; Dodd et al., 2010; Thor and Peiter, 2014). To exert this function, free Ca^2+^ levels in the cytosol under non-stimulated conditions need to be kept at a low level of around 0.1 μM. This is achieved by the action of biochemical buffers (Sanders et al., 1999) as well as H^+^/Ca^2+^ antiporters and Ca^2+^-ATPases, which actively deliver Ca^2+^ into the apoplast or intracellular stores (Dodd et al., 2010; Kudla et al., 2010, 2018). To generate a Ca^2+^ signal, the ion thus can move down the concentration gradient into the cytosol through channel proteins in the plasma or internal membranes (Figure 1).

Plants are exposed to a variety of pathogens, ranging from bacteria to fungi and oomycetes, and are also attacked by insects. To protect themselves against this biotic stress, they have evolved a two-layered immune signaling network providing defense. The first layer is termed PAMP-triggered immunity or PTI and leads to basal defense. Conserved microbial patterns (pathogen-associated molecular patterns or PAMPs) or host-derived damage-associated molecular patterns (DAMPs) are recognized by surface-localized pattern-recognition receptors (PRRs), initiating a signaling cascade leading to induction of downstream responses such as defense gene expression, stomatal closure, and callose deposition at the infection site. The Ca^2+^ signal is one of the earliest responses in this cascade (Blume et al., 2000; Dodds and Rathjen, 2010). To evade this defense, pathogens have evolved effector molecules, which are delivered into the host cell and target defense-related components. These effectors in turn are recognized by intracellular receptors [nucleotide-binding (NB)-leucin-rich repeat (LRR) receptors or NLRs] inducing the second layer of defense called effector-triggered immunity or ETI, which often is characterized by the occurrence of programmed cell death at the site of infection, the so-called hypersensitive response or HR. Ca^2+^ signals also play a role in ETI (Grant et al., 2000).

This review summarizes our current knowledge of the role calcium plays in the plant, with a focus on structural aspects, nutrition, and immunity. It describes known genes involved in its uptake and in the generation of Ca^2+^ signals and discusses possible links.

## Calcium as a Structural Component

It has been known for many years that calcium determines the rigidity of the cell wall. Ca^2+^ thereby is cross-linking negatively charged carboxyl groups of de-esterified pectin in the middle lamella. In addition to cell walls, Ca^2+^ also stabilizes cell membranes through the interaction with phospholipids (Marschner, 1995; Hepler, 2005). A low calcium content therefore weakens the cell wall, which for example in the case of pollen tubes or root hairs is necessary to allow expansion during tip growth (Bascom et al., 2018). Interestingly, the application of EGTA to growing root hairs leads to bursting of the root hairs, which however does not seem to be a consequence of the cell wall rigidity itself but rather due to the disruption of the regulatory oscillating cytosolic tip Ca^2+^ gradient, which restricts growth after every cell expansion phase (Monshausen et al., 2008). The FERONIA (FER) receptor-like kinase has been implicated in root hair development (Duan et al., 2010) and a recent publication showed that salinity stress softens the cell wall most likely due to disturbance of ionic interactions by Na^+^, which in *fer* mutants leads to defects in restoring root growth under salt stress. These defects can be rescued by treatment with calcium and borate. FER interacts with pectin, and it is suggested that it senses the wall damage and induces a Ca^2+^ signal to induce restoration of cell wall integrity (Feng et al., 2018). It was also reported that periplasmic arabinogalactan glycoproteins (AGPs) can bind Ca^2+^ and therefore be a source for cytosolic Ca^2+^ elevations during tip growth (Lamport and Várnai, 2013).

The external application of calcium has been reported to be beneficial for resistance of plants against pathogens. Tomato plants cultivated in nutrient solution with high calcium content show increased resistance against infection by *Ralstonia solanacearum* (Yamazaki et al., 2000) and increasing calcium supply to soybean plants grown in the field reduced *Phytophthora* stem rot (Sugimoto et al., 2010). In accordance with this, early studies reported a correlation of a low plant calcium tissue content with a higher fungal infection rate, suggesting that due to the lower membrane stability, the efflux of compounds such as sugars into the apoplast could be increased (Marschner, 1995). Another early study revealed the correlation between high calcium supply and the induction of defense gene expression, suggesting that this would be due to Ca^2+^ acting as second messenger (Raz and Fluhr, 1992). In addition, calcium was reported to inhibit cell wall-degrading enzymes produced by fungi and bacteria, thus a lower calcium content could lead to higher degradation and infection (Marschner, 1995). After the cuticle, the cell wall constitutes the major barrier for invading pathogens. Nowadays, the understanding of the events happening at the cell wall during pathogen attack is much more detailed. We now know that cell wall-degrading enzymes and cell wall fragments can induce signaling pathways, in which Ca^2+^ serves as second messenger. Thus, it is not only a constituent of the cell wall itself but also transduces information on cell wall damage during pathogen attack.

## Uptake and Distribution of Calcium in the Plant

Calcium is taken up from the soil solution through plasma membrane channels expressed in roots (White et al., 2002). While earlier, these channels were described based on their electrophysiological characteristics (Demidchik et al., 2002; White et al., 2002; Miedema et al., 2008), advances in the sequencing of genomes have led to the identification of different channel families and the functional characterization of specific members thereof. According to their electrophysiological properties, Ca^2+^-permeable channels in root cells were first classified into depolarization-activated channels (DACCs) and hyperpolarization-activated channels (HACCs) (Miedema et al., 2001, 2008). In addition, voltage-independent channels (VICs) have been described. Based on tissue expression, plasma membrane localization in root cells, and electrophysiological characteristics, annexins have been proposed to encode the HACCs, while members of the cyclic nucleotide-gated (CNGC) family of proteins and glutamate receptor homologs (GLRs) have been suggested to account for VIC-induced currents (Demidchik et al., 2002; White et al., 2002). However, most of these channels have been studied in the context of a potential role in signaling rather than nutrition.

As an important nutrient, calcium has to be taken up and distributed within the plant; however, its function as messenger affects the way it can be taken from the root surface to the xylem, in which it is transported to the shoot. In principle, both the apoplastic and the symplastic pathway could be available for this movement, but, as discussed earlier, Ca^2+^ concentrations in the cytosol need to be in the submicromolar range to ensure the ability of the cell to generate a Ca^2+^ signal. Symplastic movement therefore would interfere with the signaling function of calcium. A common model suggests that Ca^2+^ moves apoplastically from the epidermis through the cortex until it reaches the casparian strip of the endodermis (White, 2001). The casparian strip forms a barrier around endodermal cells, which consists mainly of suberin and lignin and prevents movement of water and solutes in order to inhibit the uptake of unwanted or toxic substances (Schreiber et al., 1999). Thus, reaching this cell layer, Ca^2+^ will have to enter the cytosol of the endodermal cells via channel proteins and be exported into the stelar apoplast via Ca^2+^-ATPases or Ca^2+^/H^+^ antiporters to finally be loaded into the xylem (Figure 1). The identities of the channels mediating the influx into endodermal cells are currently unknown. In addition to this pathway, which involves the symplast to a certain extent, a purely apoplastic pathway has been proposed (White, 2001). This could occur in regions of the root where the casparian strip of the endodermis is not yet fully developed or interrupted, such as the root apex or at the sites of lateral root emergence. An interplay between the two pathways would saturate the need of both, nutrition of and signaling in root cells as well as transport of Ca^2+^ to the xylem for distribution to the shoot (White, 2001).

Once loaded into the xylem, calcium is transported to the shoot, where it finally has to be unloaded into and distributed within the leaf cells (White and Broadley, 2003). Recently, CNGC2 (CYCLIC NUCLEOTIDE-GATED CHANNEL 2) from *Arabidopsis* has been identified as Ca^2+^ channel responsible for influx of Ca^2+^ into the leaf cells after unloading from the vasculature (Wang et al., 2017).

## Calcium as Second Messenger in Signaling Networks

While early work concentrated on the role of calcium as a nutrient and structural component, it has become more and more clear now that the main function of calcium lies in its ability to serve as a second messenger in a vast variety of physiological, developmental, and stress-related processes, and indeed some of the observed effects of calcium application might also be connected to this function rather than being purely structural.

The generation of a Ca^2+^ signal is the result of a complex interplay between influx channels and exporters as well as pumps. As calcium signals are involved in a diverse range of processes, it is assumed that the spatiotemporal pattern of the calcium rise, the so-called Ca^2+^ signature, determines the specificity of the corresponding response (McAinsh and Pittman, 2009; Dodd et al., 2010). Different sets of proteins may determine the signature in response to different cues, although some components might be involved in several responses. As important as the players generating the signal are those that decode it into (specific) downstream responses. These are calmodulin (CaM), CaM-like proteins (CMLs), calcineurin B-like proteins (CBLs), CBL interacting protein kinases (CIPKs) as well as Ca^2+^-dependent kinases (CDPKs or CPKs in *Arabidopsis*). While CaM, CMLs, CBLs, and CDPKs contain EF-hand Ca^2+^-binding motifs, CIPKs are activated by the interaction with CBLs (Kudla et al., 2018). Activation of downstream targets such as transcription factors, transporters, or channels then further relays the signal into outputs such as gene expression or stomatal closure.

In the context of this review, the focus will be on two processes, nutrient and immunity signaling.

## Nutrient Signaling

Calcium not only is a nutrient itself, interestingly in its function as second messenger, it is involved in signaling nutrient availability and changes thereof. This has been reported with relation to potassium, nitrate, iron, ammonium, and boron (Wilkins et al., 2016; Kudla et al., 2018) and also suggested for magnesium (Tang and Luan, 2017). *Arabidopsis* roots exhibit Ca^2+^ signals in response to K^+^ deficiency. The transport proteins responsible for the uptake of K^+^, AKT1 (*Arabidopsis* K^+^ transporter 1), and HAK5 (high-affinity K^+^ transporter 5) in *Arabidopsis* are both regulated via the same Ca^2+^ decoding complex consisting of CBL1/9 and CIPK23 (Xu et al., 2006; Ragel et al., 2015; Behera et al., 2017). The channels involved in the generation of the Ca^2+^ signal are still unknown. CIPK23 also regulates the activity of the transceptor IRT1 (iron-regulated transporter 1), which transports iron, but also zinc, manganese, cobalt, and cadmium. CIPK23 in this case has been shown to phosphorylate IRT1 upon binding of non-iron metals, which subsequently leads to its degradation and thus prevents unfavorable concentrations of other metals in the cytosol (Dubeaux et al., 2018). The involvement of CIPK23 thus also suggests the involvement of a Ca^2+^ signal in this process. Another nutrient transporter which is phosphorylated and thereby regulated by CIPK23 is NRT1.1 (nitrate transporter 1.1, also CHL1) (Ho et al., 2009). This phosphorylation occurs under low-nitrate conditions and turns the protein from a low- into a high-affinity transporter and downregulates primary nitrate responses (Ho et al., 2009). Despite the involvement of CIPK23 in all these processes, Ho et al. could show that there is no cross talk between potassium and nitrate sensing. In a later study, Riveras et al. (2015) could finally show a nitrate-induced, NRT1.1- and phospholipase C-dependent Ca^2+^ signal.

## Plant Immunity Signaling

In immunity signaling, the recognition of a ligand by its receptor triggers a cascade of events involving influx of Ca^2+^ into the cytosol, the production of reactive oxygen species (ROS), MAPK signaling, and expression of defense genes as well as late responses such as callose deposition and stomatal closure. Prominent examples for PAMPs or DAMPs are the bacterial flagellin, or its synthetic analog flg22, which in *Arabidopsis* is recognized by the receptor FLS2 (FLAGELLIN SENSING 2) (Zipfel et al., 2004) or the endogenous peptide signal AtPep1 (Huffaker et al., 2006), which is recognized by PEPR1 and PEPR2 (Yamaguchi et al., 2006, 2010). The activity of pathogenic enzymes also leads to the production of cell wall fragments, such as oligogalacturonides (OGs). OGs result from the breakdown of pectin by polygalacturonases and serve as DAMPs to induce defense signaling. In *Arabidopsis*, they are recognized by the receptor Wall Associated Kinase 1 (WAK1) (He et al., 1996; Brutus et al., 2010). Cell wall-degrading enzymes can also serve as PAMPs themselves.

The influx of Ca^2+^ constitutes one of the earliest responses in the induced cascades. The channels mediating the Ca^2+^ influx are so far mostly unknown. Mainly two families of channels have been investigated in this context, CNGCs and GLRs. For example, mutants in the *AtCNGC2* locus were entitled “defense, no death” (*dnd1*) according to their phenotype, which showed no HR but still resistance to avirulent *Pseudomonas syringae* as well as a range of virulent pathogens (Yu et al., 1998). *dnd1* mutants exhibit an autoimmune phenotype, being dwarf and showing constitutively elevated levels of salicylic acid (SA) as well as defense gene expression such as *PR1* (Yu et al., 1998; Clough et al., 2000). Wang et al. (2017) recently found that the *dnd1* phenotype is dependent on the calcium supply in the medium. Consistent with a previous report by Chan et al. (2003), they found that the dwarf appearance of the mutant was dependent on high calcium supply, as was the suppression of HR. It was thus suggested that in the mutant, the CNGC2-mediated unloading of Ca^2+^ into the leaf cells is interrupted, leading to higher apoplastic calcium which then is strengthening the cell wall, thereby preventing HR. The establishment of the hypersensitive response as part of the plant defense therefore in this case seems to be depending on the nutritional status of the plant, i.e., the nutritional status of the plant with regard to calcium is influencing its immunity. CNGC2 has also been implicated in lipopolysaccharide-induced induction of nitric oxide (NO) production required for HR (Ali et al., 2007) as well as signaling upon perception of Pep3, but not flg22 (Ma et al., 2012). *atglr3.3* mutants showed higher susceptibility to *Hyaloperonospora arabidopsidis* and were impaired in OG-induced NO and ROS production as well as gene expression; however, a reduction of the Ca^2+^ signal in these plants could not be shown (Manzoor et al., 2013). Recently, GLR3.3 and GLR3.6 together with TPC1 (two-pore channel1) have been shown to be involved in the generation of local cytosolic Ca^2+^ elevations at aphid feeding sites. These elevations were also shown to be dependent on the coreceptor BAK1 with the corresponding receptor perceiving the herbivore-associated molecular pattern still awaiting identification (Vincent et al., 2017).

On the decoding side, several CDPKs have been shown to be part of plant immunity signaling. Silencing of *NtCDPK2* resulted in impairment of gene-for-gene-dependent HR (Romeis et al., 2001). Multiple knockout mutants of *CPK4*, *CPK5*, *CPK6*, and *CPK11* are compromised in flg22-induced production of ROS, resistance to *Pst* DC3000, and show reduced levels of expression of a subset of flg22-induced genes in PTI signaling (Boudsocq et al., 2010). CPK5 has also been shown to phosphorylate respiratory burst oxidase homolog protein D (RBOHD) and participate in long-distance signaling of defense responses (Dubiella et al., 2013). In addition, *cpk5/6/11* mutants are more susceptible to infection with *Botrytis cinerea* due to defects in ethylene production (Gravino et al., 2015). In NLR-mediated immunity, CPK4/5/6/11 phosphorylate a specific set of WRKY transcription factors to induce immune gene activation (Gao et al., 2013). CPK5 has recently been assigned a unique role in plant immunity in that, in contrast to CPK4, 6, or 11, it contributes to *exo70B1*-activated autoimmune responses via association with the truncated NLR protein TIR-NBS2 (TN2) (Liu et al., 2017). Another Ca^2+^-dependent kinase, CPK28, has been shown to be a negative regulator of PTI signaling (Monaghan et al., 2014).

## Perspective

Calcium is an essential element in plants. It serves as a constituent of cell walls and membranes and thus contributes to the structure of cells and the upholding of physical barriers against pathogens. In accordance with this structural role, plants deficient in calcium have been shown to be more susceptible to pathogens and exogenous calcium supply in turn has been shown to improve the plant’s resistance. However, in recent years, it has emerged that the main function of calcium is to serve as a second messenger. It does so in signaling events connected to a vast variety of physiological, developmental, and environmental cues, among them the signaling of other nutrients as well as pathogen attack. Research is now concentrating on identifying the proteins mediating influx of Ca^2+^ in as well as export out of the cell and those that decode Ca^2+^ signals into downstream responses. Some genes have already been identified and their role in specific processes described; however, most of the proteins mediating Ca^2+^ uptake and distribution as well as signal generation and decoding are still unknown. Also, despite few examples, surprisingly little is known about how the involvement of calcium in one plant aspect is affecting another and whether the same or different genes are involved in two processes, as, for example, those taking up Ca^2+^ as a nutrient in roots and those responsible for influx during signal generation. Future research will shed more light on the different functions of this important nutrient and how they are interconnected.

## Author Contributions

The author confirms being the sole contributor of this work and has approved it for publication.

### Conflict of Interest Statement

The author declares that the research was conducted in the absence of any commercial or financial relationships that could be construed as a potential conflict of interest.
